# Supplementary material for: Oxidative switch drives mitophagy defects in dopaminergic parkin mutant patient neurons
Source: Sci Rep. 2020 Sep 23;10:15485. doi: 10.1038/s41598-020-72345-4 (PMC7511396; doi:10.1038/s41598-020-72345-4)
Supplement: Supplementary file 1 — Supplementary Figure 1. [file 41598_2020_72345_MOESM1_ESM.docx]

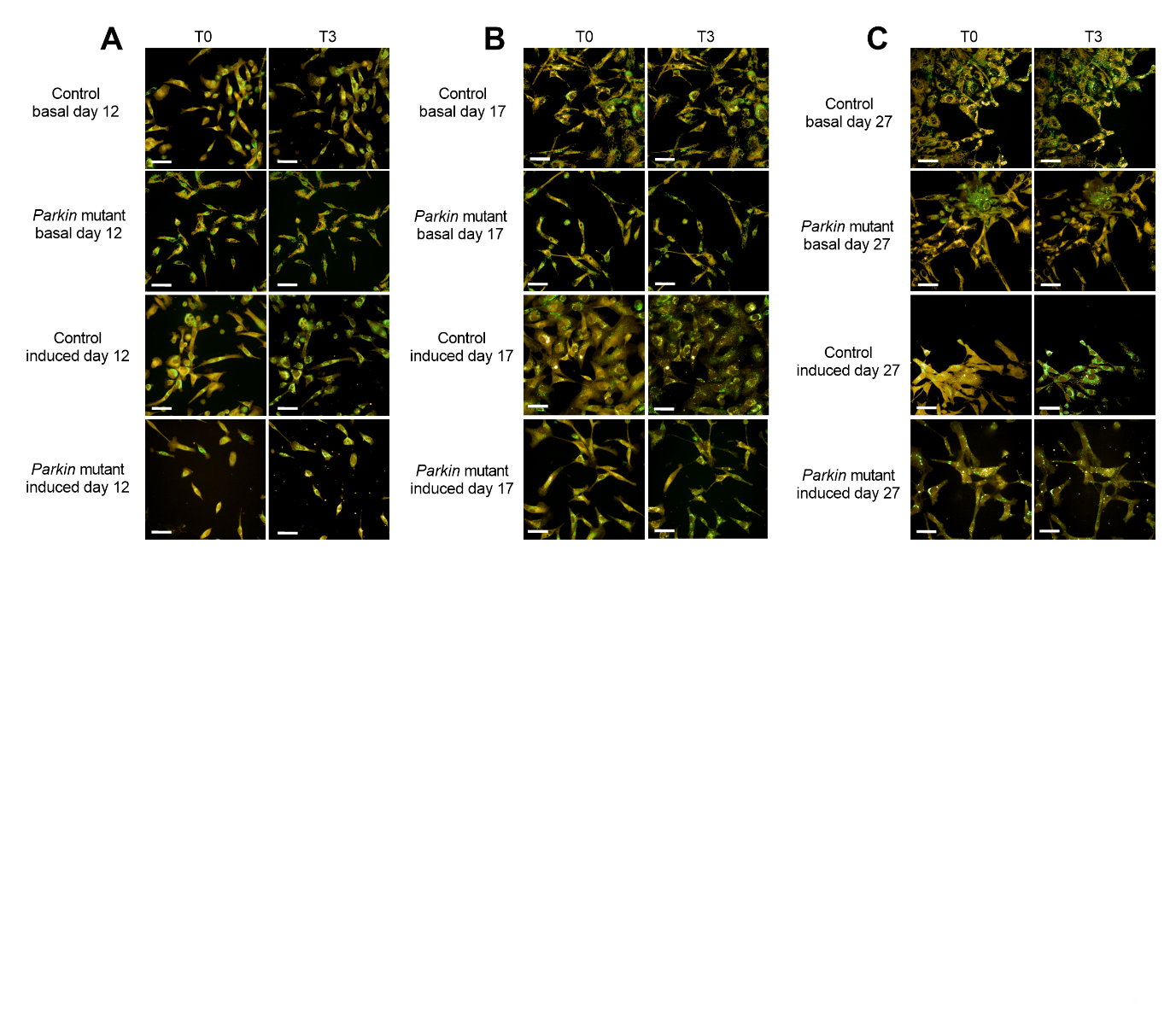


Supplementary Figure Legends. Supplementary Figure 1. Representative images from the live mitophagy assay throughout differentiation. A representative image is shown from a control line and a PRKN mutant at 12 days differentiation (A), 17 days differentiation (B) and 27 days differentiation (C) at two time points T0 and T3. Images are shown for basal and induced mitophagy (induced with mitochondrial inhibitors antimycin A and oligomycin).
